# Haptoglobin Genotype-Dependent Anti-Inflammatory Signaling in CD163^+^ Macrophages

**DOI:** 10.1155/2013/980327

**Published:** 2013-04-23

**Authors:** R. Clive Landis, Pandelis Philippidis, Jan Domin, Joseph J. Boyle, Dorian O. Haskard

**Affiliations:** ^1^Edmund Cohen Laboratory for Vascular Research, Chronic Disease Research Centre, The University of the West Indies Bridgetown BB11115, Barbados; ^2^Eric Bywaters Centre for Vascular Inflammation, Faculty of Medicine, Imperial College London, London W12 0NN, UK; ^3^Department of Life Sciences, University of Bedfordshire, Luton LU1 3JU, UK

## Abstract

Intraplaque hemorrhage causes adaptive remodelling of macrophages towards a protective phenotype specialized towards handling iron and lipid overload, denoted Mhem. The Mhem phenotype expresses elevated levels of hemoglobin (Hb) scavenger receptor, CD163, capable of endocytosing pro-oxidant free Hb complexed to acute phase protein haptoglobin (Hp). It is notable that individuals homozygous for the Hp 2 allele (a poorer antioxidant) are at increased risk of cardiovascular disease compared to the Hp 1 allele. In this study, we examined whether scavenging of polymorphic Hp:Hb complexes differentially generated downstream anti-inflammatory signals in cultured human macrophages culminating in interleukin (IL)-10 secretion. We describe an anti-inflammatory signalling pathway involving phosphatidylinositol-3-kinase activation upstream of Akt phosphorylation (pSer473Akt) and IL-10 secretion. The pathway is mediated specifically through CD163 and is blocked by anti-CD163 antibody or phagocytosis inhibitor. However, levels of pSer473Akt and IL-10 were significantly diminished when scavenging polymorphic Hp2-2:Hb complexes compared to Hp1-1:Hb complexes (*P* < 0.05). Impaired anti-inflammatory macrophage signaling through a CD163/pAkt/IL-10 axis may thus represent a possible Hp2-2 disease mechanism in atherosclerosis.

## 1. Introduction

Intraplaque hemorrhage is a common complication of atherosclerosis and is linked to plaque progression, especially in diabetes [1–4]. However, work from independent groups has demonstrated that macrophages at zones of hemorrhage may exert some level of homeostatic control through adaptive remodelling towards an Mhem phenotype capable of handling iron and lipid overload [5–7]. Scavenging of haptoglobin:hemoglobin (Hp:Hb) complexes via CD163 is part of this adaptive process, linked to secretion of anti-inflammatory cytokine interleukin (IL)-10 and elevation of heme oxygenase (HO)-1 [8–10]. Analogous protective pathways are evoked by free diffusion of purified heme or by phagocytosis of damaged erythrocytes via CD204, leading to the proposal that a final common pathway is instigated by accumulation of intracellular heme capable of transcriptionally activating genes involved in iron handling and cholesterol efflux (e.g., HO-1 and liver X receptor) via transcription factors Nrf2 and activating transcription factor (ATF)-1 [11, 12]. Although the atheroprotective properties of the Mhem macrophage phenotype is therefore well established, the role of proximal signalling pathways linked to anti-inflammatory IL-10 secretion via CD163-dependent uptake of Hb:Hp remains to be fully understood.

In addition to IL-10, immunoregulatory IL-6 cytokine has been consistently reported downstream of CD163 [8, 13, 14]. However, the earliest IL-6 studies using cross-linking anti-CD163 antibodies may not have adequately discriminated between surface versus endocytosis-dependent effector pathways [13, 14]. Whether downstream signals require simple cross-linking of CD163 at the cell surface or phagocytosis of the entire Hp:Hb complex is an important distinction, since Hp2-2:Hb binds more avidly than Hp1-1:Hb to CD163 at the surface but, conversely, is more poorly internalised into the cell [15, 16]. More recent investigations employing native Hp:Hb ligand appeared to suggest poor or even lack of dependence on CD163 for IL-6 or IL-10 signalling pathways, depending on the type of polymorphic haptoglobin variant employed [17, 18]. Since the haptoglobin 2 allele is linked to a host of adverse clinical cardiovascular events, [19–23] it is important to understand Hp genotype-dependent disease mechanisms in CD163^+^ macrophages in greater detail, to guide informed interdictions in vulnerable individuals.

Here we have examined IL-10 signalling pathways during scavenging of polymorphic Hp2-2:Hb versus Hp1-1:Hb complexes in CD163^+^ human monocyte-derived macrophages. We identify a specific Akt/IL-10 pathway that is comparatively underinduced during the scavenging of Hp2 complexes.

## 2. Materials and Methods

### 2.1. Reagents and Antibodies

Human Hb (A_o_), human Hp (phenotypes 1-1 and 2-2), and colchicine were purchased from Sigma-Aldrich (Poole, UK). Anti-human CD163 monoclonal antibody clones RM3/1, Ki-m8, and 5C6-FAT were purchased from Bachem (Merseyside, UK), clone GHI/61 from BD Pharmingen (Oxford, UK), and clone Ber-MAC3 from Dako (Cambridge, UK). Polyclonal anti-Akt and anti-phosphoAkt (Ser473) antibodies were purchased from Cell Signalling Technology, Inc. (Beverley, MA). The phosphoinositide-3-kinase (PI-3K) inhibitor, Ly294002, was purchased from Alexis Corporation (Bingham, UK). Endotoxin determinations were made using the GCL-1000 LAL chromogenic endpoint assay (Cambrex Bio Science, Wokingham, UK).

### 2.2. Hb:Hp Treatment of Macrophage Cultures

Human monocytes were isolated from venous blood and differentiated into CD163^+^ macrophages *in vitro* as described [8]. Hb:Hp complexes were generated by dissolving equimolar amounts of Hb and Hp in growth medium. Hb, Hp, or Hb:Hp were added at final concentrations of 1 mg/mL unless otherwise stated to monocyte/macrophage cultures prior to incubation for 24 hours and collection of supernatants and/or cell lysates for IL-10 and Akt analysis, respectively. Hb or Hp batches containing detectable endotoxin (>5 pg/mL) were discarded. Supernatants and cell lysates were stored in aliquots at −70°C prior to analysis. In some experiments, the PI-3K inhibitor Ly294002 was added at 50, 25, 12.6, and 6.25 *μ*mol/L final concentrations. Actinomycin D and cycloheximide were added at a concentration of 1 *μ*g/mL and colchicine at a concentration range between 10 and 1.25 *μ*mol/L. None of the inhibitors at the concentrations used exhibited significant macrophage cellular cytotoxicity [24]. Anti-CD163 monoclonal antibodies were added at a final concentration of 20 *μ*g/mL, previously shown to be neutralizing [8].

### 2.3. Enzyme Linked Immunosorbent Assays

IL-10 concentrations in culture supernatants were determined by ELISA technique (Quantikine; R&D Systems, Abingdon, UK) according to the manufacturer's recommendations. Phospho-Akt levels in cell lysates, collected in buffer consisting of 1% Triton X-100, 25 mmol/L sodium deoxycholate, 150 mmol/L NaCl, 50 mmol/L Tris pH 7.4, 4 mmol/L EDTA, 200 *μ*mol/L sodium orthovanadate, 10 mmol/L sodium pyrophosphate, 100 mmol/L sodium fluoride, 1 mmol/L phenylmethylsulfonyl fluoride, and 5% protease inhibitor cocktail (Sigma Aldrich), were measured by pSer473 Akt kit (Biosource, Camarillo, CA) according to manufacturer's instructions. All samples were measured in duplicate and results expressed as mean cytokine concentration (pg/mL) or pAkt activity (U/mg) ± SEM from *n* = 3 experiments.

### 2.4. Western Blot Analysis

Monocytes/macrophages were lysed in buffer, as described for the phospho-Akt ELISA, and proteins were separated by SDS-PAGE on a 12.5% gel prior to transfer to Immobilon-P membranes (Millipore Corporation, Bedford, MA). Equal loading of lanes was confirmed by estimation of lysate protein content using the Bio-Rad D_c_ protein assay (Bio-Rad, Hercules, CA). Induction of pSer473Akt was established relative to total Akt by probing blots with polyclonal antibody against pSer473Akt, followed by stripping and reprobing with total Akt. Blots were developed with an enhanced chemiluminescence substrate (Amersham Pharmacia Biotech UK Ltd, Little Chalfont, UK). 

### 2.5. Statistical Analysis

Statistical comparisons between Hp1-1:Hb and Hp2-2:Hb groups were performed using an unpaired Student's *t*-test. Multiple group comparisons of pSer473Akt activity were performed using a one way ANOVA with a Student-Newman-Keuls posttest. Statistical analysis was performed using GraphPad Prism (GraphPad Prism Software, Inc., San Diego, CA), and significance was assumed at *P* < 0.05.

## 3. Results

### 3.1. Impaired IL-10 Secretion following CD163-Dependent Scavenging of Hp2-2:Hb

**Figure 1:**
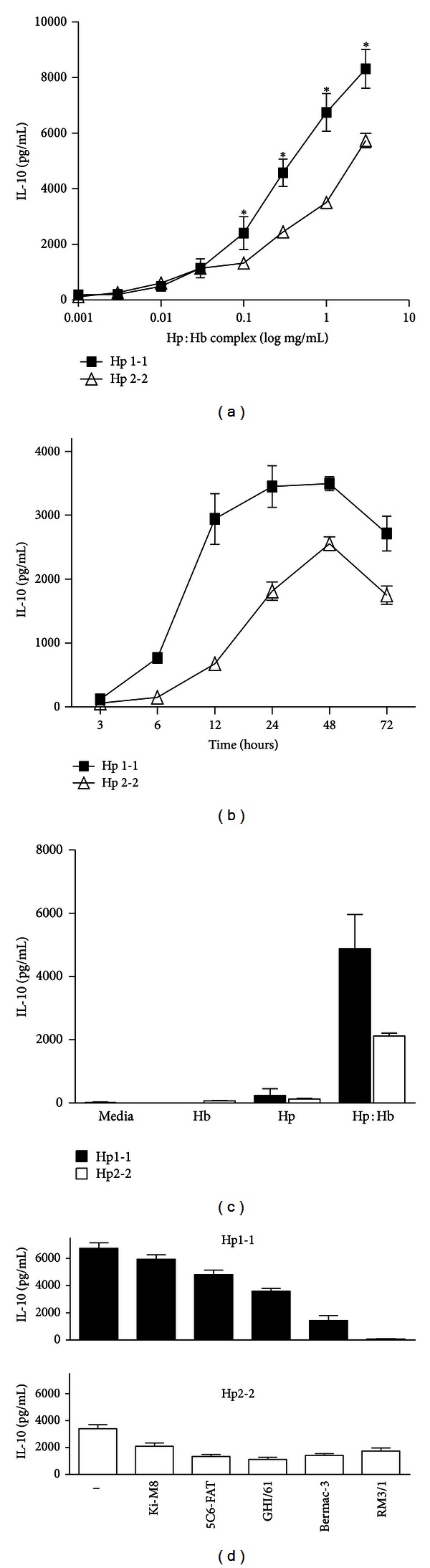
CD163-dependent IL-10 secretion following scavenging of polymorphic Hp:Hb complexes. (a) Effect of increasing concentrations of polymorphic Hp:Hb complexes on IL-10 secretion by CD163^+^ human monocyte-derived macrophages at 24 h. (b) Timecourse of IL-10 secretion following addition of polymorphic Hp:Hb complexes at 1 mg/mL. (c) IL-10 secretion by CD163^+^ macrophages stimulated with Hb alone, Hp1-1, Hp2-2, or polymorphic Hp:Hb complexes (1 mg/mL) at 24 h. (d) Effect of a panel of anti-CD163 antibodies (20 *μ*g/mL) on IL-10 secretion induced by polymorphic Hb:Hp complexes (1 mg/mL) at 24 h. All IL-10 assays were carried out in duplicate, and results are expressed as mean IL-10 (pg/mL) ± SEM from *n* = 3–9 experiments.  *P < 0.01.

IL-10 induction has been linked to CD163 receptor engagement [8, 13, 14, 17, 18]. Differential IL-10 responses by CD163^+^ macrophages were therefore examined in the presence of polymorphic Hp:Hb complexes. The addition of Hp:Hb complexes across a range of concentrations to *in vitro* differentiated CD163^+^ macrophages revealed significantly impaired IL-10 induction in the case of Hp2-2. At concentrations within the range of plasma haptoglobin (0.1–2 g/L) [18, 25], the IL-10 response to Hp2-2:Hb was significantly diminished compared to Hp1-1:Hb (e.g., at 1 g/L: 3509 ± 169 pg/mL versus 6739 ± 678, *P* < 0.01; Figure 1(a)). The timecourse of IL-10 induction to either type of Hp:Hb complex was relatively slow, with a peak at 48 h (Figure 1(b)). Consistent with the slow kinetic, IL-10 secretion required prior transcription and protein synthesis, as it was abrogated in the presence of actinomycin D or cycloheximide (data not shown). In agreement with previous observations [8, 15, 17] and again repeated here, IL-10 induction required formation of a Hp:Hb protein complex, since neither Hb alone, nor Hp alone yielded significant IL-10 secretion (Figure 1(c)). A panel of five anti-CD163 antibodies showed a similar inhibitory profile to either type of complex with the notable exception of the function blocking anti-CD163 antibody RM3/1 [8]. RM3/1 abolished IL-10 secretion in response to Hp1-1:Hb but only weakly inhibited the response to Hp2-2:Hb (Figure 1(d)).

### 3.2. Signaling Pathways Linked to CD163-Dependent Hp:Hb Scavenging

**Figure 2:**
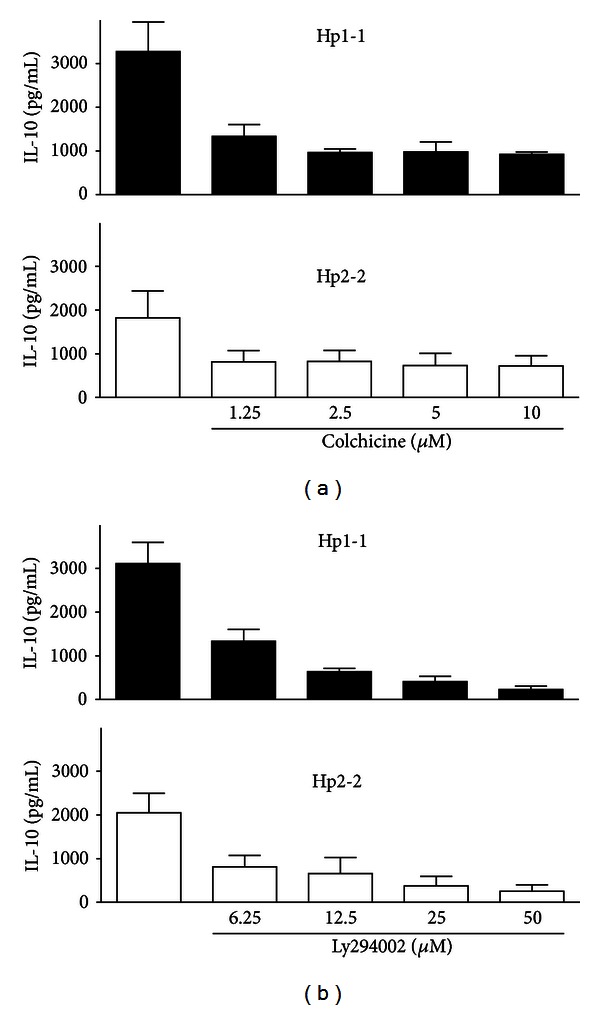
Effect of pharmacological inhibitors on Hp dependent IL-10 secretion. Effect of (a) colchicine (1.25–10 *μ*mol/L) and (b) Ly294002 (6.25–50 *μ*mol/L) on IL-10 release by CD163^+^ human monocyte-derived macrophages stimulated with polymorphic Hp:Hb complexes (1 mg/mL) for 24 h. Results are expressed as mean IL-10 (pg/mL) ± SEM from *n* = 3–4 experiments.

The IL-10 response to either type of Hp required phagocytosis of complexes, since it was blocked in the presence of the phagocytosis inhibitor colchicine (Figure 2(a)). A screen of PI-3K, PKC, p42/44 MAP kinase, and p38 MAP kinase signaling pathway inhibitors revealed that the IL-10 response to Hp:Hb complex scavenging was dose dependently inhibited by the PI-3K inhibitor LY294002 (Figure 2(b)). This was true for Hp2-2:Hb as well as Hp1-1:Hb complexes. The pan-PKC inhibitor bisindolylmaleimide, the p42/44 MAP kinase inhibitor U0126, and p38 MAP kinase inhibitor SB203580, used at a concentration range known to inhibit cytokine secretion in the same cell preparation against calcific microcrystals [24], had no effect on IL-10 secretion (data not shown).

**Figure 3:**
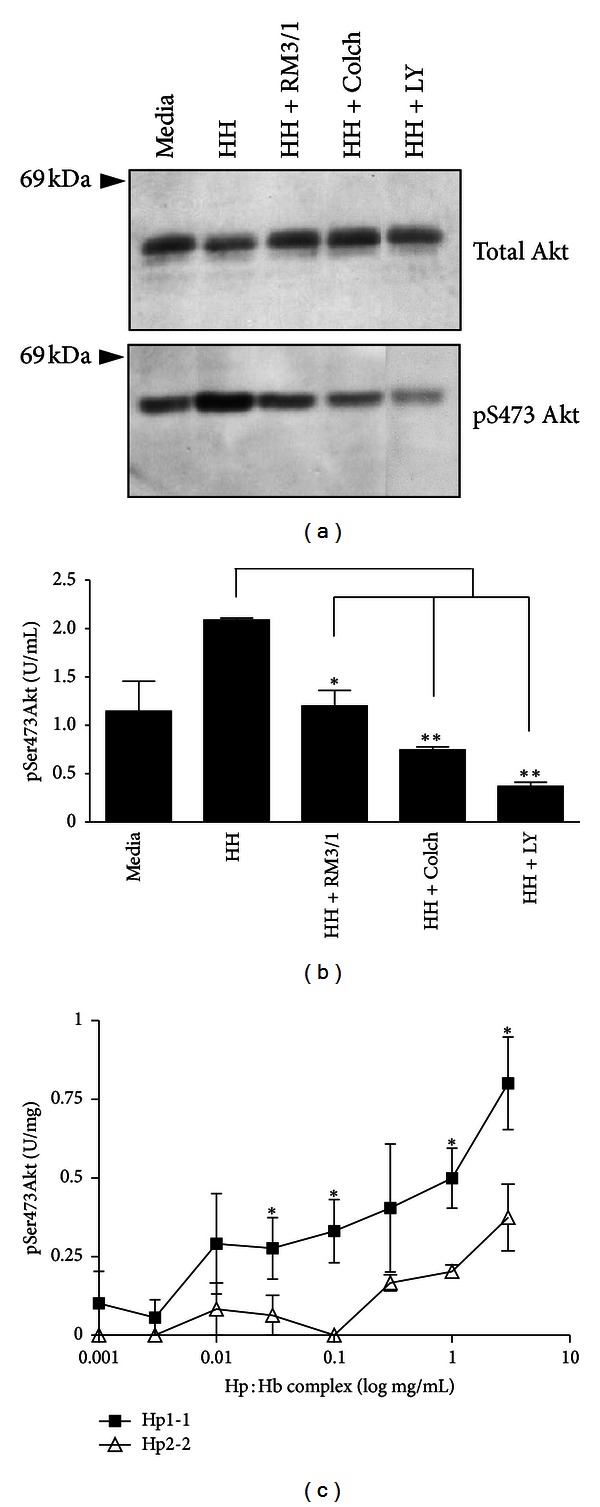
Hp:Hb scavenging-dependent Akt phosphorylation. (a) Representative Western blot showing phosphorylated Akt (pSer473) and total Akt in lysates from macrophages stimulated with Hp1-1:Hb (1 mg/mL; HH in legend) for 20 minutes in the presence and absence of blocking anti-CD163 antibody clone RM3/1 (20 *μ*g/mL; RM3/1 in legend), colchicine (2.5 *μ*mol/L; colch in legend), or phosphoinositol 3-kinase inhibitor Ly294002 (12.5 *μ*mol/L; Ly in legend). (b) Quantitative ELISA determination of Akt (pSer473) phosphorylation in cell lysates from macrophages treated as in (a) above. (c) Comparison of Hp2-2:Hb versus Hp1-1:Hb scavenging on Akt phosphorylation in cell lysates, as measured by pSer473Akt-specific ELISA. Results in (b) and (c) are expressed as pSer473Akt (U/mg) ± SEM from three experiments.  *P < 0.05,  **P < 0.01.

To further explore signaling pathways linked to CD163 downstream of PI-3 kinase, phosphorylation of Akt was investigated. Phosphorylation of Akt at serine 473 (pSer473Akt) was examined by Western blot as well as phosphoSer473Akt-specific ELISA: both assays showed phosphorylation of Ser473Akt and this was inhibited significantly by anti-CD163 antibody RM3/1 or colchicine (Figures 3(a) and 3(b)). Akt phosphorylation required upstream PI-3Kinase activation, as it was abrogated in the presence of PI-3Kinase inhibitor Ly294002 (Figures 3(a) and 3(b)). Akt phosphorylation was markedly impaired following scavenging of Hp2-2:Hb complexes compared to Hp1-1:Hb, across a broad concentration range (e.g., at 1 g/L: 0.20 ± 0.02 U/mg versus 0.50 ± 0.10, *P* < 0.05; Figure 3(c)).

## 4. Discussion

The central role of Hp in complexing free Hb and mediating its clearance through CD163 led us to consider whether the Hp2-2 genotype, clinically associated with cardiovascular complications, may show impaired anti-inflammatory signaling engagement downstream of CD163. The model chosen for this work (human monocytes differentiated for 7 days in culture) benefits from the absence of exogenously added differentiating agents (e.g., steroids) and has been validated against human macrophages differentiated *in vivo*: equivalent functional responses to Hp:Hb scavenging were noted in CD163^+^ macrophages recovered from human skin blisters during the cutaneous inflammatory response to cantharidin [8]. The CD163/Akt/IL-10 axis identified here adds to a growing understanding that homeostatic signalling pathways may be triggered in CD163^+^ macrophages during hemoglobin scavenging and that these are impaired in the case of polymorphic Hp2-2 protein. Our data add a cellular dimension to the innate antioxidant properties recognised for Hp variants (Hp1-1 superior to Hp2-1 superior to Hp2-2) [4, 26, 27] and suggest a plausible new disease mechanism linking Hp2-2 with atherothrombosis. 

Our findings are consistent with previous reports on the relative inability of Hp2-2:Hb complexes to trigger IL-10 responses [17, 18] and may explain why previous reports noted an apparent lack of CD163 receptor usage by polymorphic Hp2-2:Hb variants [17, 18]. In those studies, RM3/1 was employed as the blocking antibody. Here we confirm that RM3/1 is the most potent blocking antibody against Hp1-1:Hb induced IL-10 secretion, out of a screen of five anti-CD163 antibodies (RM3/1, Ki-M8, 5C6-FAT, GHI/61, and Bermac-3). However, RM3/1 exhibited only weak blocking against Hp2-2:Hb complexes. Hence, it is not likely that Hp2-2:Hb signals through a different (unknown) receptor pathway. The evidence presented here with other blocking antibodies (Bermac3 and GHI/61) as well as the phagocytosis inhibitor colchicine strongly suggests that both types of Hp:Hb complexes are endocytosed via CD163.

Results from the panel of blocking antibodies also have implications for the way in which signals are generated through CD163. The elegant structure-function analysis of CD163 protein [28], which combined ligand binding studies in solution with a comprehensive epitope map of ten anti-CD163 antibodies, failed to detect any direct ligand-blocking property for RM3/1. Only Ki-m8 and Edhu-1, mapping to the ligand binding scavenger receptor cysteine-rich (SRCR) domain 3, directly inhibited Hp:Hb binding to CD163 in solution. Since these same two antibodies behaved as agonistic antibodies when added to cells [8, 13, 14], this supports the concept that cross-linking of two or more ligand-binding domains on CD163 must be required for signal generation, as originally proposed [15]. Out of the panel of ten anti-CD163 antibodies previously mapped, RM3/1 uniquely mapped to the last SRCR domain (domain 9, immediately proximal to the plasma membrane) [28]. This suggests that its function blocking property relates to an ability to block dimerization of CD163 at the membrane following cross-linking with native Hp1-1:Hb holo-dimer. Since Hp2-2 exists as a larger multimer, consisting of 3–8 subunits, this might explain why RM3/1 only partially blocks IL-10 signal production in response to the multimeric complex. 

We describe a hitherto unappreciated signalling pathway following Hp:Hb complex endocytosis, requiring Ser473Akt phosphorylation and culminating in IL-10 secretion. We recognise the limitations posed through the use of a pharmacologic inhibitor of PI-3 kinase, but phosphorylation of Ser473Akt was verified both at Western blot and quantitative intracellular ELLISA levels. Adenoviral infection approaches to further dissect the signalling pathway were not successful, since infection even with empty vectors triggered phosphorylation of Akt, therefore ruling out this approach (data not shown). Past studies that used adenovirus to investigate signalling pathways in macrophages used differentiating agents not compatible with the CD163^+^ endpoint and our cells may therefore have behaved differently [29].

Studies carried out prior to the identification of Hp:Hb as ligand for CD163 employed a cross-linking anti-CD163 antibody, EDhu-1, and reported IL-6 secretion that was casein kinase II and PKC dependent [13, 14]. Secretion of IL-6 in our hands, in response to Hp:Hb, was not specific to CD163, since it did not require Hp complex formation with Hb, did not require an intact microtubular assembly for phagocytosis, and was not blocked by function-blocking antibodies or a PI-3K inhibitor (data not shown). Our own results do not therefore support IL-6 as an effector molecule downstream of CD163, whereas IL-10 was confirmed and was Hp genotype dependent. Alternative receptor mechanisms and endotoxin contamination were excluded as confounding sources of intracellular signals. Mac-1 was eliminated as a possible alternative candidate receptor for haptoglobin [30]. Endotoxin levels in all cell cultures were below 5 pg/mL and inhibition of IL-10 secretion by phagocytic inhibitors also ruled out endotoxin as a possible trigger. Our previous work has shown that above a threshold concentration of >10 ng/mL of Hp:Hb complexes, all or none commitment to the CD163^+^ phenotype occurs via an IL-10/CD163 feedback loop [31]. This scenario is consistent with observations that CD163^+^ macrophages closely associate with areas of hemorrhage [10, 31].

In summary, the present study has identified an PI-3/Akt signaling pathway contributing to IL-10 secretion via CD163^+^-dependent hemoglobin scavenging, a pathway that is impaired during the scavenging of polymorphic Hp2-2 complexes. This adds not only to our understanding of cardiovascular disease susceptibility for Hp2-2 but may also be relevant to the protection of the vasculature and kidneys from heme-mediated oxidative injury secondary to hemolysis [32–35], especially in predisposing conditions like diabetes, sickle cell anemia, or leukaemia treatment [16, 36, 37]. In conjunction with previous observations in atherosclerotic plaques [5, 12, 31], we propose that CD163^+^ macrophages may act as a homeostatic brake on plaque inflammation secondary to hemorrhage, in an Hp genotype dependent manner.
